# XMRV: usage of receptors and potential co-receptors

**DOI:** 10.1186/1743-422X-8-423

**Published:** 2011-09-06

**Authors:** Mohan Kumar Haleyur Giri Setty, Krishnakumar Devadas, Viswanath Ragupathy, Veerasamy Ravichandran, Shixing Tang, Owen Wood, Durga Sivacharan Gaddam, Sherwin Lee, Indira K Hewlett

**Affiliations:** 1 Center for Biologics Evaluation and Research, Food and Drug Administration, Bethesda, MD 20892, USA

## Abstract

**Background:**

XMRV is a gammaretrovirus first identified in prostate tissues of Prostate Cancer (PC) patients and later in the blood cells of patients with Chronic Fatigue Syndrome (CFS). Although XMRV is thought to use XPR1 for cell entry, it infects A549 cells that do not express XPR1, suggesting usage of other receptors or co-receptors.

**Methods:**

To study the usage of different receptors and co- receptors that could play a role in XMRV infection of lymphoid cells and GHOST (GFP- Human osteosarcoma) cells expressing CD4 along with different chemokine receptors including CCR1, CCR2, etc., were infected with XMRV. Culture supernatants and cells were tested for XMRV replication using real time quantitative PCR.

**Results:**

Infection and replication of XMRV was seen in a variety of GHOST cells, LNCaP, DU145, A549 and Caski cell lines. The levels of XMRV replication varied in different cell lines showing differential replication in different cell lines. However, replication in A549 which lacks XPR1 expression was relatively higher than DU145 but lower than, LNCaP. XMRV replication varied in GHOST cell lines expressing CD4 and each of the co- receptors CCR1-CCR8 and bob. There was significant replication of XMRV in CCR3 and Bonzo although it is much lower when compared to DU145, A549 and LNCaP.

**Conclusion:**

XMRV replication was observed in GHOST cells that express CD4 and each of the chemokine receptors ranging from CCR1- CCR8 and BOB suggesting that infectivity in hematopoietic cells could be mediated by use of these receptors.

## Introduction

A new gamma retrovirus, Xenotropic Murine leukaemia Virus-related virus (XMRV), was identified in 2006 and its association was claimed with prostate cancer (PC) and chronic fatigue syndrome (CFS). A series of studies from disparate geographical areas have failed to substantiate these claims. Recent studies have suggested that XMRV may have arisen in mice through recombination of two proviruses [1].

Regardless of the controversies, XMRV is a culturable virus capable of infecting different cell types like T and B Lymphocytes, NK cells, etc., [2]. Intravenous inoculation of Rhesus Macaques with XMRV showed organ-specific cell tropism, infecting CD4 T cells in lymphoid organs including the gastrointestinal lamina propria, alveolar macrophages in lung, and epithelial/interstitial cells in other organs, and cells of the reproductive tract [3]. Many retroviruses are pathogenic (HIV-1) causing severe disease but at the same time they have been used in gene therapy which requires targeting of the virus to the host cell through interactions between viral envelope proteins and cell surface proteins. It is important to determine the mode of its cell entry to define tropism, and understand virus transmission and pathogenesis. Biological process involves highly specific interactions and infection of cells by viruses is no exception. Such interactions are of major importance in pathogenic viruses as they are potential drug targets. One such classic example is the entry of HIV-1 through a series of interactions between the viral gp120 and cellular receptor CD4 and co-receptor such as either CCR5 or CXCR5 [4].

The xenotropic/polytropic subgroup of mouse leukemia viruses (MLVs) all rely on the XPR1 receptor for entry, but these viruses vary in tropism, distribution among wild and laboratory mice, pathogenicity, strategies used for transmission, and sensitivity to host restriction factors [5]. XMRV is closely related to xenotropic murine leukemia viruses MLVs (X-MLVs) [6]. The X-MLVs and polytropic MLVs (P-MLV) use Xpr1 as a receptor for cell entry [4,7,8], and so does XMRV [9-11]. The recent identification of MLV and XMRV in human prostate cancer tissues, peripheral blood mononuclear cells (PBMCs) of chronic fatigue syndrome patients, and the respiratory tract of immunocompromised patients [12] raises the concern of a potential threat to public health from cross-species transmission of MLV related viruses.

## Results and discussion

**Figure 1:**
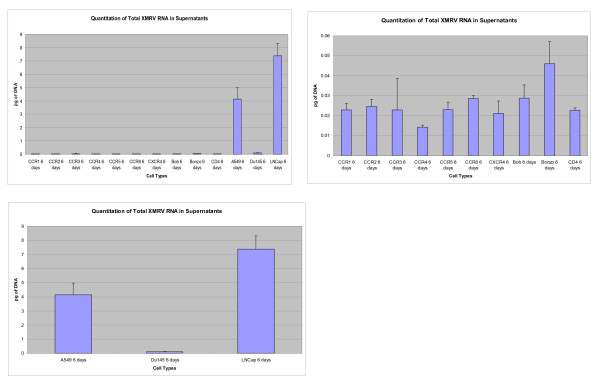
**Quantitation of XMRV RNA in 6 day infected culture supernatant**. Figure 1(A)- GHOST cell lines expressing different Receptors. Figure 1(B)- Lung epithelium A549 cell line; Prostate cancer cell lines DU 145 and LNCap. Figure 1(C) Comparison of Ghost Cell lines expressing different receptors with A549, DU145 and LNCap which support high replication of XMRV.

**Figure 2:**
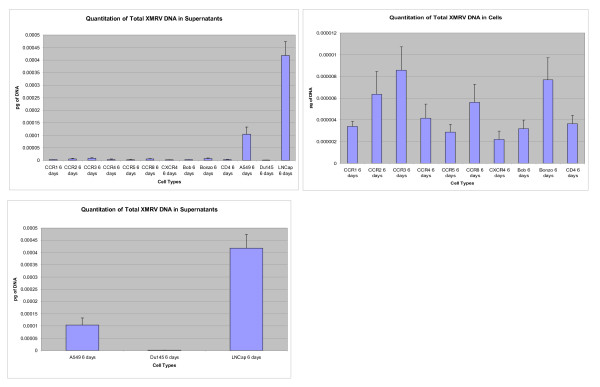
**Quantitation of XMRV DNA in 6 day infected cultured Cells**. Figure 2(A)- GHOST cell lines expressing different Receptors. Figure 2(B)- Lung epithelium A549 cell line; Prostate cancer cell lines DU 145 and LNCap. Figure 2(C) Comparison of Ghost Cell lines expressing different receptors with A549, DU145 and LNCap which support high replication of XMRV.

Infection and replication of XMRV was observed in a variety of GHOST cells, LNCaP, DU145, A549 and CaSki cell lines (Figure 1 and 2). The levels of XMRV replication varied in different cell lines. However, replication in A549 which lacks XPR1 expression was relatively high compared with DU145 but lower than levels observed in LNCaP (Figure 1C and 2C). These findings suggest that perhaps other molecules could serve as receptors for this virus in addition to Xpr1. XMRV replicated less efficiently in GHOST cell lines expressing CD4 and each of the co- receptors CCR1 - CCR8 and Bob compared with A549 and LNCaP. However, among the GHOST cell lines there was significant replication of XMRV in CCR3 and Bonzo expressing cells (Figure 1C and 2C).

XMRV infection of GHOST cells that express CD4 and each of the chemokine receptors ranging from CCR1- CCR8 and Bob suggests that infection and tropism for hematopoietic cells could be mediated by use of these receptors as XPR1 is not expressed in blood cells and very weakly expressed in bone marrow (Gene Atlas). Replication of XMRV in A549 cells lacking XPR1 was much higher when compared with DU145 which express XPR1. Furthermore, the cell line 293T expresses XPR1 but did not support XMRV replication. These findings clearly indicate the possibility of other receptors for XMRV than XPR1. Interestingly CCR1, CCR2, and CCR3 are expressed in A549 and Bonzo in PC-3, DU145, LNCaP and A549 [13,14]. More research may be needed to fully understand the scope and extent to which this mouse derived virus uses other receptors to enter human cells and cross species susceptibility in line with its xenotropic nature.

## Materials and methods

### Cells

GHOST (GFP- Human osteosarcoma) cells expressing CD4 along with different chemokine receptors including CCR1, CCR2, etc., were grown in Dulbecco's modified Eagle's medium (DMEM Quality Biologics) with 10% fetal bovine serum (FBS).

### XMRV infectivity in various cell types

After three and six days of infection the XMRV levels in supernatant and the integrated viral DNA in cells were quantified using real time quantitative PCR. The values were normalized by taking equal amounts of RNA/DNA and compared with XMRV clone VP62-pcDNA3.1 standard (GenBank accession no. EF185282; obtained through NIH AIDS Research and Reference Reagent Program).

### RNA isolation

Total RNA and DNA were isolated using Qiagen extraction kits.

### Quantitative PCR

An equal amount of total RNA was used to quantify XMRV levels using QuantiTect Probe RT-PCR kit (Qiagen). Real-time PCR Master Mix (Quantitect Probe RT-PCR, Qiagen) was added with forward and reverse primers, probe, and cDNA or RNA in a total volume of 25 uL. Real-time PCR from RNA was done with the same contents as above in addition to RT-mix (Qiagen) and primer for Viral RNA. The mixture was incubated at 50°C for 2 min (for RNA, 20 min), at 95°C for 10 min, and then cycled at 95°C for 15 sec and 60°C for 60 sec 40 times, using the Applied Biosystems 7500 sequence detection system.

## Competing interests

The authors declare that they have no competing interests.

## Authors' contributions

All authors read and approved the final manuscript. MH performed experiments; MH, IKH and KD contributed to designing research, analyzing data, discussing findings, and writing the manuscript; KD, SL and OW virus culture and propagation; ST virus copy number determination; ST, VR, DSG and VR contributed to Real-time PCR primer design and assay standardization; OW and VR GHOST cell culture and propagation. IKH supervised the work.
